# Correlation of blood-based immune molecules with cardiac gene expression profiles reveals insights into Chagas cardiomyopathy pathogenesis

**DOI:** 10.3389/fimmu.2024.1338582

**Published:** 2024-02-08

**Authors:** Thaiany G. Souza-Silva, Eula G. A. Neves, Carolina Koh, Andrea Teixeira-Carvalho, Silvana Silva Araújo, Maria do Carmo Pereira Nunes, Juliana de Assis Silva Gomes, Kenneth J. Gollob, Walderez Ornelas Dutra

**Affiliations:** ^1^ Laboratório Biologia das Interações Celulares, Departament de Morfologia, Instituto de Ciências Biológicas, Universidade Federal de Minas Gerais, Belo Horizonte, MG, Brazil; ^2^ Rene Rachou Institute, Fundação Oswaldo Cruz (FIOCRUZ), Belo Horizonte, MG, Brazil; ^3^ Clínica Médica, Faculdade de Medicina, Universidade Federal de Minas Gerais, Belo Horizonte, MG, Brazil; ^4^ Hospital Israelita Albert Einstein, São Paulo, SP, Brazil; ^5^ Instituto Nacional de Ciências e Tecnologia em Doenças Tropicais, Belo Horizonte, Brazil

**Keywords:** Chagas Cardiomyopathy, circulation, gene expression profiling, T cells, inflammation

## Abstract

**Introduction:**

Understanding compartmentalized immune responses in target organs is crucial for elucidating the pathogenesis of various diseases. However, obtaining samples from affected vital organs often poses safety challenges. In this study, we aimed to investigate potential correlations between the levels of disease-associated immune molecules in the bloodstream with their gene expression profiles in the hearts of patients suffering from Chagas Cardiomyopathy (CCC). This debilitating and often fatal condition is caused by infection with the protozoan Trypanosoma cruzi.

**Methods:**

Blood samples were analyzed using the Bio-Plex platform. Gene Expression Omnibus (GEO) database was used to determine gene expression profile in heart tissue from CCC and non-Chagas controls (CTRL).

**Results:**

Elevated levels of inflammatory cytokines were detected in the plasma of CCC patients, and these levels correlated with clinical indicators of deteriorating cardiac function. Notably, 75% of the soluble factors assessed in the plasma exhibited a consistent relationship with their gene expression levels in the cardiac tissue of CCC patients. Analysis of interactions and signaling pathways related to these molecules revealed an overrepresentation of inflammatory pathways in both blood and heart compartments. Moreover, we identified that differentially expressed genes in CCC cardiac tissue were primarily associated with T-cell signaling pathways and correlated with the presence of CD8+ T cells in the myocardium.

**Discussion:**

Our findings establish a strong correlation between relevant immune molecules and their signaling pathways in both the blood and heart tissue in CCC. This validates the use of blood as a non-invasive medium for understanding immunopathology and identifying markers for cardiac dysfunction in Chagas disease.

## 1 Introduction

Despite the significance of dissecting compartmentalized immune responses to elucidate cell-cell interactions and disease mechanisms, accessing targeted organs can be challenging due to safety considerations. Therefore, establishing a correlation between immune molecules detected in the blood and their expression in the affected organ provides invaluable insights into localized immunopathology and aids in identifying disease markers non-invasively.

Chagas disease, triggered by *Trypanosoma cruzi* infection, affects over 6 million people and 100 million individuals are at risk of infection (1). While the majority of chronically infected individuals remain asymptomatic in the indeterminate form (IND), approximately 30% progress to the cardiac clinical form (CCC), characterized by ventricular dilation with impaired function leading to heart failure, arrhythmias, thromboembolism and ultimately, death (2, 3). CCC is considered one of the most debilitating and deadliest cardiomyopathies (1, 4).

Understanding why some patients remain asymptomatic while others develop severe cardiac disease, as well as the mechanisms that control the progression of cardiomyopathy, remains a challenge. However, it is clear that the immune response plays a pivotal role in these processes. Several studies by our group and others have shown that while human CCC is associated with the predominant expression of inflammatory cytokines and chemokines, IND has been associated with a more balanced response (5–10). These studies clearly show the importance of immunoregulatory processes in the development and progression of Chagas disease. Most studies on Chagas disease have focused on peripheral blood, where activation of T-cells, indicated by the expression of activation markers, cytokines and by proliferative response, has been observed in both IND and CCC patients (reviewed by Koh et al., (11)). Additionally, the presence of T-cells expressing cytotoxic markers and cardiotropic chemokine receptors has been detected in the peripheral blood of Chagas patients (12, 13). While T-cells, inflammatory cytokines and cytotoxic molecules have also been found in the myocardium of CCC (14–16), their functional characteristics and role in disease pathogenesis are poorly understood. Recent studies have explored transcriptional profiles of human left ventricular free wall heart tissue samples from CCC patients, revealing differences in DNA methylation and microRNA expression (17–20). However, an integrative transcriptomic approach investigating the association with immune cells and their functions is still lacking.

Obtaining cardiac tissue from CCC patients is challenging, as heart biopsies are not typically recommended. Moreover, heart tissue acquired from CCC patients undergoing transplantation primarily represents an advanced disease stage. This is also an issue with many other diseases with localized pathology. Hence, peripheral blood remains a valuable and non-invasive source of information for understanding disease progression and identifying biomarkers of severity.

Here, we aimed to establish a correlation between the levels of blood-derived immune factors and the gene expression for these molecules in the heart of CCC patients. In addition, we performed network and signaling pathway analysis in the two compartments, identifying mechanisms related to cellular responses and immunoregulation in CCC. Our results showed a clear correspondence of the expression of several immune factors between blood and heart, identifying biological pathways relevant to disease pathology, and consolidating blood as a valid and reliable material for investigating disease pathogenesis and biomarker discovery in Chagas disease.

## 2 Patients and methods

### 2.1 Study population

This cross-sectional study used plasma samples of 84 individuals to measure circulating molecules. These individuals were divided in the following groups: (1) patients with CCC (CCC; n= 40) with positive serology for *Trypanosoma cruzi*, left ventricular enlargement accompanied by impaired systolic function and previous or current symptoms of heart failure (3); (2) patients in the indeterminate clinical form (IND; n= 32) of Chagas disease, with positive serology for *T. cruzi* and without clinical manifestation related to Chagas disease (21); (3) healthy donors (CTRL; n= 12), with negative serology for *T. cruzi* and normal ventricular function. The patients are from the estate of Minas Gerais, and were recruited at the outpatient clinic of the Hospital das Clínicas at the Federal University of Minas Gerais, where they are examined, characterized, and receive all care indicated by the responsible clinician and team. Exclusion criteria included: diabetes mellitus, thyroid dysfunction, renal insufficiency, chronic obstructive pulmonary disease, rheumatic heart disease, as well as any autoimmune disease or chronic inflammatory disorders. The demographic and clinical data of groups are represented in the **Supplementary Table 1**.

Quantification of left ventricular size and function were obtained in accordance to the recommendations of the American Society of Echocardiography guidelines (22). Left ventricular diastolic diameter (LVDD) was measured at the end-diastole in the long-axis view, and left ventricular ejection fraction (LVEF) was derived by calculating the variance between end-diastolic and end-systolic volumes and then dividing it by the “end-diastolic volume”. We have expanded our analysis to encompass not only patients with Chagas cardiomyopathy but also those in the indeterminate phase of the disease. Correlation of cytokine expression with LVEF and LVDD from IND did not present any statistical significance, likely because these patients have normal left ventricular dimension with preserved LVEF. This additional information has been included in **Supplementary Table 1** of the **Supplementary Material**.

We performed a second set of experiments measuring a higher number of molecules, as described below. This confirmatory experiment was performed in a subset of patients following the same clinical characterization described above, from the same region and recruited and cared for at the Hospital das Clínicas (UFMG). For this set, we enrolled 17 CTRL, and 30 CCC patients.

All individuals were informed about the objectives of study and signed the Informed Consent Form. Research Ethics Committee of Universidade Federal de Minas Gerais (COEP 93968218.7.0000.5149) approved this study.

Gene expression profile analysis included transcripts of human left ventricular free wall heart tissues from end-stage heart failure CCC patients (n= 10) and healthy individuals (CTRL, n= 7), using the accession number GSE84796 at the publicly available Gene Expression Omnibus (GEO) database (https://www.ncbi.nlm.nih.gov/geo/).

### 2.2 Soluble factors measurement

Blood from all patients was obtained and used as a source of plasma, following protocol routinely used by us (23). Briefly, blood was collected in vacutainers containing heparin as anticoagulant. Material was processed within 4 hours after collection, submitted to centrifugation (10min, 400g at room temperature) for removal of plasma. Plasma was aliquoted and stored in cryotubes at -80 °C. Levels of soluble molecules were evaluated in the plasma of the different study groups using CBA assay (BD™ Human Th1/Th2/Th17 CBA; Cat # 560484). Data were acquired by the FACScantoII, according to manufacturer’s instructions and results expressed as the mean fluorescence intensity (MFI). The second set of experiments was performed using the 48-plex (Bio-Plex Pro ™ Human Cytokine Screening Panel; Cat #12007283) and read in the Bio-Plex 200 instrument equipped with the Manager software and the results were expressed as MFI.

### 2.3 Differentiation pattern of study groups

The pattern of differentiation of soluble cytokines and chemokines between groups was analyzed using Clustvis software (R-version 0.7.7 package). Heat map analysis allows to identify clusters within the sample and to identify homogeneity or heterogeneity in the distribution of soluble molecules between the groups. Rows and columns are grouped using the correlation distance and the mean link. Principal component analysis (PCA) was performed, and the X and Y-axes show the percentage of the total variance. Prediction ellipses indicates a probability of 0.95 that a new observation will fall inside the ellipse (24).

### 2.4 Microarray data and processing

Gene Expression Omnibus (GEO) database (https://www.ncbi.nlm.nih.gov/geo/) was used to determine gene expression profile in heart tissue from CCC and CTRL. GEO2R (http://www.ncbi.nlm.nih.gov/geo/geo2r/) web tool was used for comparing the two groups. The p-values using Benjamin and Hochberg false discovery rate method were applied in order to correct the occurrence of false positive results (25). An adj. P<0.05 and a logFC ≥ 1 were set as the cut-off criteria. ExpressAnalyst algorithm was used for visualization of functional categories enriched in a network (26).

### 2.5 Gene list collection and pathways enrichment analysis

Official gene symbol from NCBI was used as the identifier for CCC-related genes. The downregulated and upregulated genes list was used for the pathway enrichment analysis by PathfindeR package on RStudio software (version 4.2.1). The Kyoto Encyclopedia of Genes and Genomes (KEGG) analyses were available at the PathfindeR package (27). The NetworkAnalyst.ca. was used to construct interaction network of differentially expressed genes (DEGs). Pairwise correlation prediction was determined based on IMEx database. High-scoring genes were used to identify hub genes. Enriched pathway analyses obtained as a result of interconnections in the network were generated using KEGG. Biological processes (BP) pathways enriched were measured by NetworkAnalyst.ca.

To predict the correlation between DEGs in CCC and immune system cells, gene clusters were used as input to the Correlation Network Hub Prediction (CNHP) tool of the Immuno-Navigator database (sysimm.ifrec.osac.osaka-u.ac.jp/immune-navigator/) (28).

### 2.6 Statistical analysis

Comparisons between plasma levels of soluble molecules between the study groups and correlation analysis were performed using the GraphPad Prism (version 8.0.2) software (GraphPad Software, La Jolla – CA, USA). Parametric data were submitted to the one-way ANOVA multiple tests followed by the Tukey post-test and represented by the mean and standard deviation. Nonparametric data were analyzed using the Kruskal-Wallis test, followed by Dunn’s post-test. Correlation analysis between plasma cytokines and chemokines with left ventricular ejection fraction (LVEF) or left ventricular diastolic diameter (LVDD) parameters were performed using Pearson or Spearman test, based on parametric or non-parametric data, respectively.

## 3 Results

### 3.1 A high correspondence of cytokine and chemokine expression was observed between blood and heart of CCC patients as compared to non-Chagas individuals

**Figure 1:**
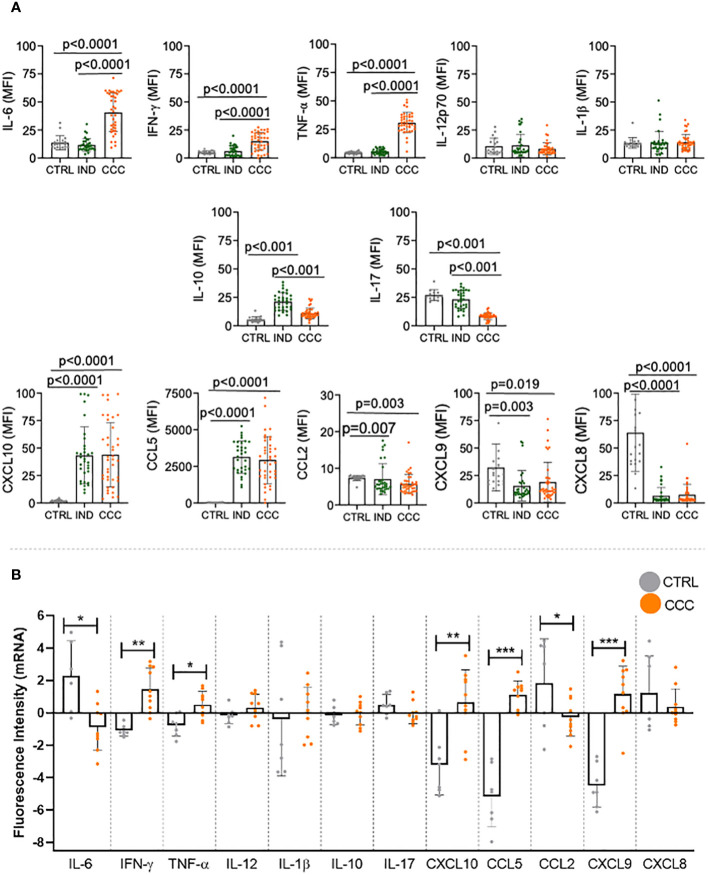
Plasma levels of cytokines and chemokines in healthy controls (CTRL), indeterminate (IND) and cardiac (CCC) Chagas patients. **(A)** Plasma soluble mediators were measured using the Bio-Plex ProTM Human Cytokine Standard 27-plex kit and results were expressed in MFI. p values < 0.05 were considered statically significant. **(B)** Differentially expressed genes of human left ventricular free wall heart tissue from CCC and CTRL (GSE84796). Data were analyzed for normality and represented as mean ± SD. P-values ≤ 0.05 were considered statistically significant. *p value = 0.05; **p value = 0.01; *** p value < 0.001.

We evaluated the levels of cytokines and chemokines in the plasma from individuals with different clinical forms of Chagas disease. First, we compared the expression of the soluble factors amongst the different clinical groups. Our data demonstrated an increase in inflammatory cytokines IL-6, IFN-γ and TNF-α in CCC compared to IND and to CTRL (**Figure 1A**). There were no differences in serum levels of IL12p70 and IL-1β between the distinct clinical forms of Chagas disease or comparing patients with CTRL (**Figure 1A**). While IL-10 expression was higher in IND as compared to CCC and CTRL, no significant change was observed comparing CCC and CTRL (**Figure 1A**). A decrease in IL-17 plasma levels was observed in CCC compared to IND and CTRL (**Figure 1A**). Regarding chemokines, CXCL10 and CCL5 were increased in IND and CCC patients compared to CTRL, while CCL2, CXCL9 and CXCL8 were reduced in both clinical forms of Chagas disease compared to CTRL (**Figure 1A**).

We then compared the expression of each molecule between CCC and CTRL in each compartment. In the blood, we found that levels of IL-6, IFN-γ, TNF-α, CXCL-10, CCL-5 were higher in CCC as compared to CTRL; levels of IL-12, IL-1β and IL-10 were similar when comparing CCC and CTRL; levels of IL-17, CCL-2, CXCL-9 and CXCL-8 were lower in CCC than CTRL. We then evaluated the gene expression for these factors in the heart of CCC patients as compared to CTRL. Our data showed that IFN-γ, TNF-α, CXCL10, CCL-5 and CXCL-9 genes were upregulated in CCC as compared to CTRL, while no significant changes were observed in the expression of IL-12, IL-1β, IL-10, IL-17 and CXCL-8 genes; CCL2 and IL-6 were downregulated in CCC as compared to CTRL (**Figure 1B**). Thus, we observed a similar behavior in blood and heart off CCC compared to CTRL in 9 out 12 molecules: IFN-γ, TNF-α, CXCL-10 and CCL-5 increased in blood and heart of CCC as compared to CTRL; IL-12, IL-1β and IL-10 levels were similar in CCC and CTRL in blood and heart; CCL-2 decreased and CXCL-8 had a clear tendency of decreased expression in blood and heart from CCC as compared to CTRL. These data show a similar behavior in 10 out of 12 molecules (~80%) in blood and heart of CCC compared to CTRL, and a statistically significant equivalence (67%) between expression of immune molecules in blood and heart tissues. A second set of experiments were performed, increasing the number of molecules analyzed. Our data showed that, of the 43 molecules that we had information in plasma and heart tissue (5 of them did not have available the gene expression in heart), 27 (63%) had a similar behavior in blood and heart, and 23 had statistically significant corresponding changes (54%) (**Supplementary Table 2**). This data confirms the high correspondence of the molecules between blood and heart, even considering a higher number of molecules analyzed.

**Figure 2:**
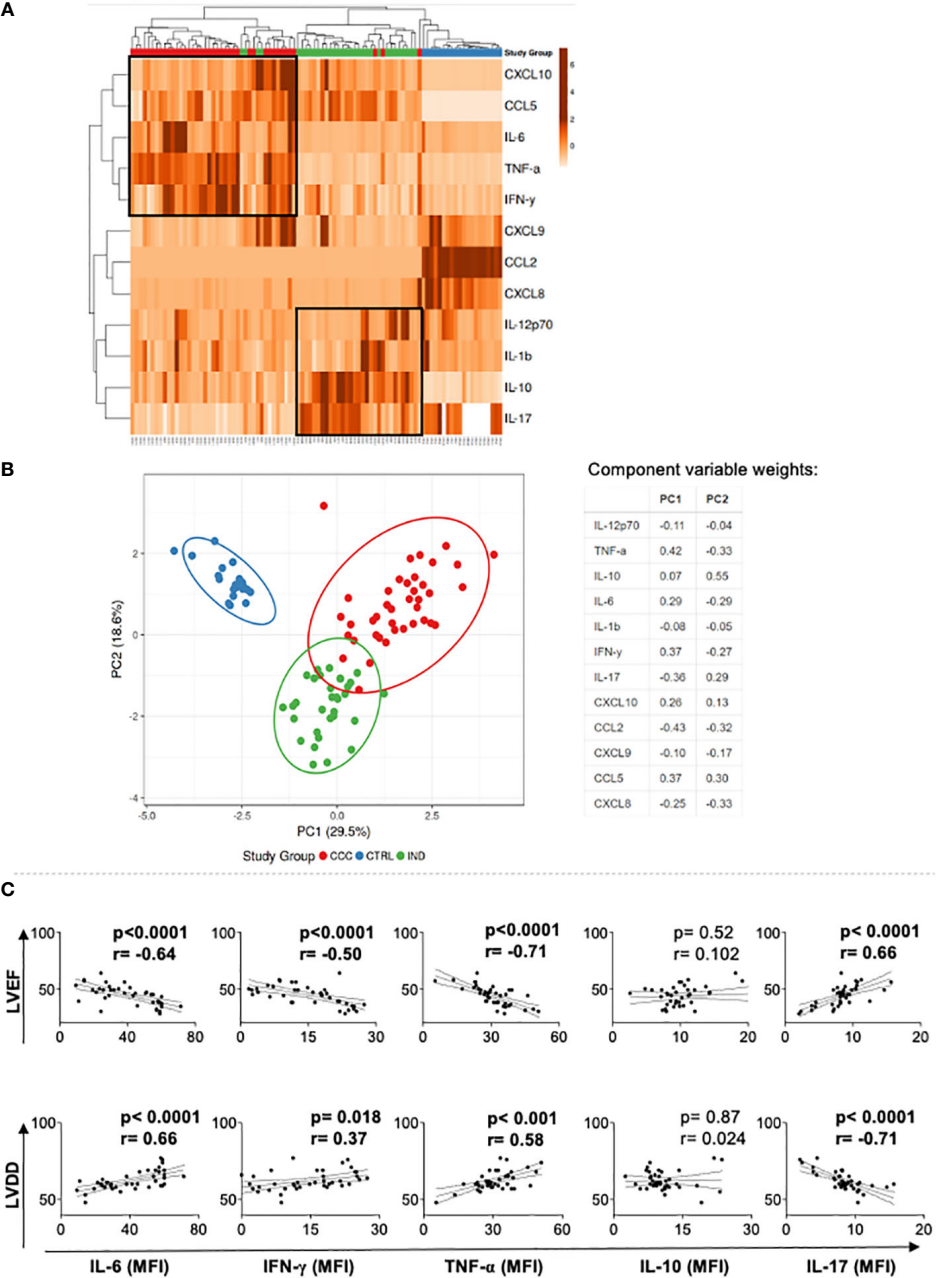
Multivariate analysis of soluble mediators in Chagas patients and healthy individuals, and correlation with markers of cardiac function. **(A)** Heatmap analysis of soluble mediators. Rows and columns were grouped using correlation distance and mean link. In the color gradient bar, light orange indicates lower plasma levels, while dark orange denotes higher levels. Vertical lines represent each donors evaluated, and horizontal lines represent each molecule in the study. The red, green, and blue blocks correspond to the CCC, IND and CTRL groups, respectively. Associations between molecules or patients are indicated by the brackets. **(B)** Principal component analyses (PCA) between measured cytokines and evaluated study groups; the X and Y axes show % of the total variance. Ellipses indicate a probability of 0.95 that a new observation will fall within the ellipse. The table shows the variable weights for each component. **(C)** Correlation analysis between cytokine plasma levels and LVEF and LVDD in Chagas cardiomyopathy patients (CCC). Parametric data were analyzed using Pearson’s correlation test and non-parametric data using Sperman’s test. p values < 0.05 were considered statically significant. LVEF, Left ventricular ejection fraction; LDVV, Left ventricular diastolic dysfunction.

To evaluate if the expression of these soluble mediators in the blood allowed for discrimination between the groups of Chagas disease patients, we performed heatmap and PCA analysis. Heatmap and PCA data showed clear segregation between CTRL and Chagas patients, and a segregation with a few overlaps between CCC and IND groups (**Figures 2A, B**). A strong correlation was observed between TNF-α, IFN-γ, IL-6, and CCL5 in CCC (**Figure 2A**), while a strong correlation between IL-10 and IL-17, IL-12 and IL-1beta was observed in IND (**Figure 2A**).

### 3.2 Soluble inflammatory cytokines are correlated with clinical markers of worse cardiac function

To determine the association between cytokine plasma levels with clinical parameters of heart dysfunction, left ventricular ejection fraction (LVEF) and left ventricular diastolic diameter (LVDD), we performed a correlation analysis between these variables in CCC group. We observed an inverse and statistically significant correlation between the inflammatory cytokines TNF-α, IL-6 and IFN-γ with LVEF, a worse prognosis predictor in Chagas disease (3), while IL-17 plasma level was positively correlated with LVEF and LVDD, indicating better heart function (**Figure 2C**). In addition, TNF-α, IL-6 and IFN-γ plasma levels were positive and statistically significant correlated with LVDD (**Figure 2C**), reinforcing that the levels of systemic inflammatory cytokines are correlated with worse heart function.

### 3.3 Blood and heart-derived mediators are correlated with enrichment of inflammatory signaling pathways

**Figure 3:**
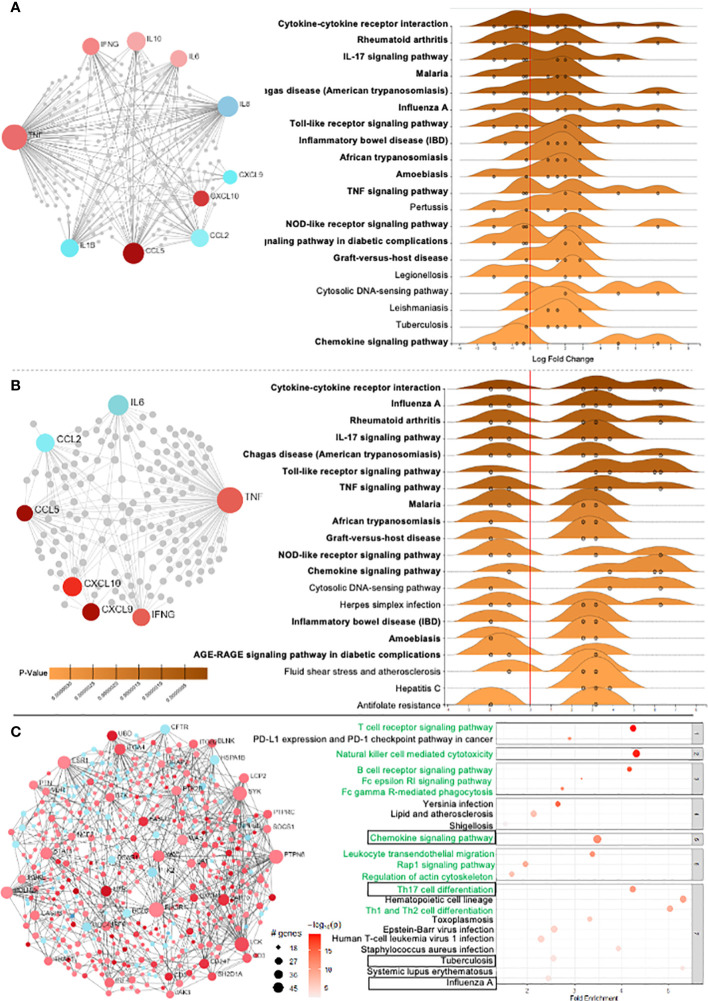
Differentially expressed genes (DEG) and network analysis in Chagas cardiomyopathy patients (CCC). **(A)** Network of 10 soluble molecules and **(B)** 7 DEG in the heart tissue in CCC patients in relation to control group. Light red nodes represent less significant and dark red more significant expression amongst the upregulated genes. Blue nodes represent significantly downregulated genes, with dark and light colors representing higher and lower variability. In the right side, the enriched KEGG pathways in the blood and heart tissue from CCC are presented. (p adj< 0.05) - GSE84796). **(C)** Network analysis of the overall DEG in the heart of CCC in relation to control group, and the corresponding signaling pathways associated to the observed interactions.

The circulating cytokines and chemokines that were differentially regulated in CCC were used to build a connectivity network and key enriched signaling pathways. Network analysis shows that inflammatory and regulatory cytokines present in the plasma of CCC compared to CTRL are intrinsically connected (**Figure 3A**). In addition, we found that soluble mediators differentially modulated in CCC as compared to CTRL mainly contribute to the enrichment of inflammatory signaling pathways such as: (i) cytokine-cytokine receptor interaction, (ii) IL-17 signaling, (iii) Chagas disease, (iv) Toll-like receptors (TLR), (v) TNF signaling and (vi) chemokine signaling (**Figure 3A**).

Genes that were differentially expressed in the heart of CCC as compared to CTRL and were concordant to expression in blood were included in the network and signaling pathway analysis, which also showed intrinsic connection between the analyzed molecules (**Figure 3B**). Similar to observed in blood, signaling pathway analysis also showed an enrichment of inflammatory pathways in the heart (**Figure 3B**). Fifteen out of the 20 enriched signaling pathways were similar between blood and heart (marked in bold in **Figures 3A, B**).

To verify the overall profile of interactions and signaling pathways in the heart, and gain insight into *in situ* immunological events, analysis of the GSE84796 dataset for all differentially expressed genes (DEG) in CCC as compared to CTRL was performed. 2166 DEGs were identified, which included 1562 upregulated and 604 downregulated genes (**Figure 3C**, red and blue dots, respectively). Of the 20 top enriched signaling pathways derived from all DEGs, 10 are directly linked to inflammatory pathways (**Figure 3C**, marked in green). Strikingly, despite the large number of DEGs included in this overall analysis, 20% of the top pathways were similar to the ones observed in blood (**Figure 3C**, marked with boxes), showing a clear biological correspondence between blood- and heart-derived molecule pathways in human Chagas disease.

To obtain insights into biological processes associated with the differentially expressed molecules in blood and heart tissue of CCC, we performed gene ontology enrichment analysis. We found that biological processes related to activation of the immune response and inflammatory responses were enriched in blood (**Supplementary Figure 1A**) and heart analysis (**Supplementary Figure 1B**). Importantly, of the top 15 biological processes, 10 were similar between blood and heart (**Supplementary Figure 1A**, marked in bold).

### 3.4 Differently expressed genes in CCC heart tissue are correlated with T lymphocytes, particularly the CD8+ subset

**Figure 4:**
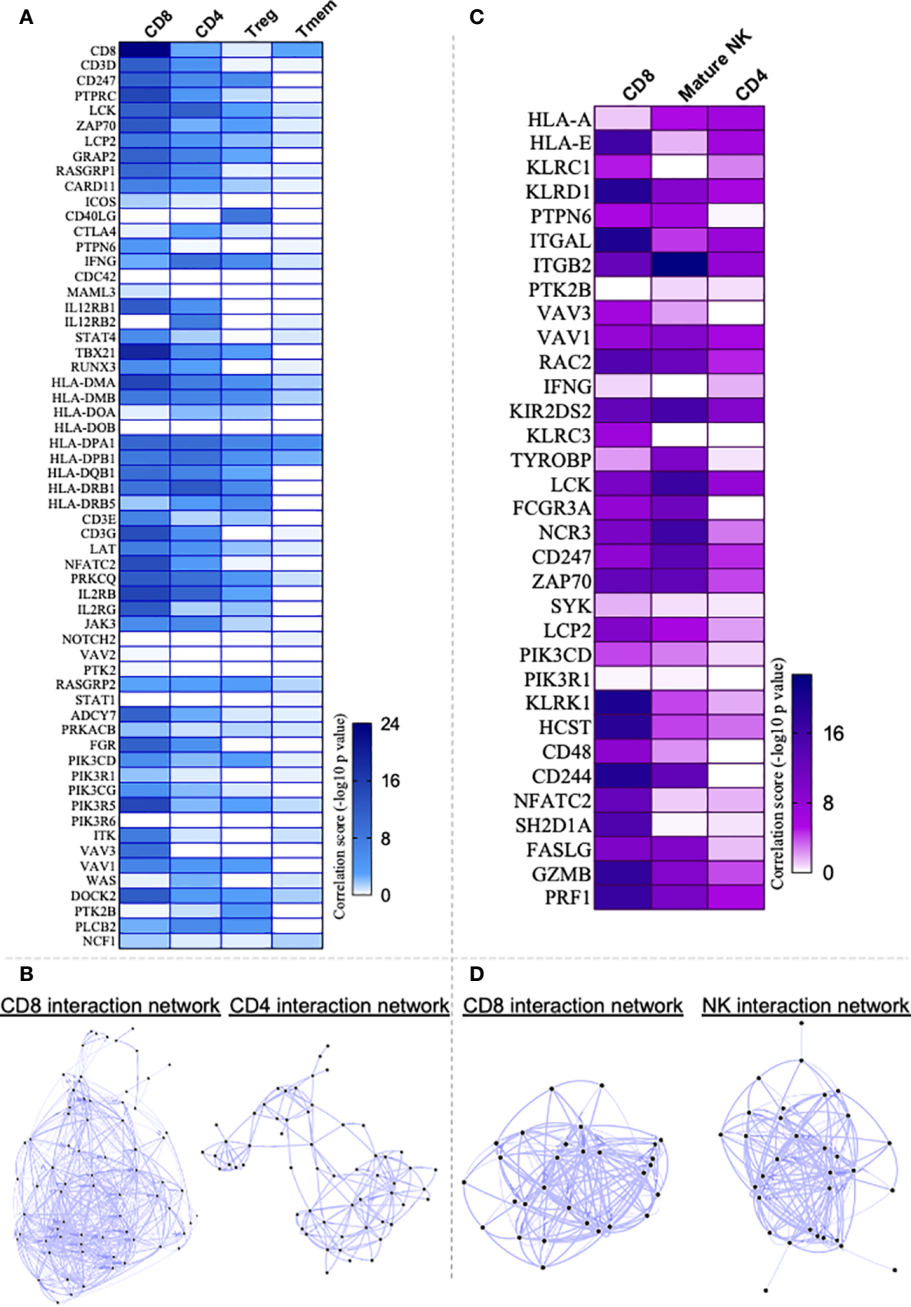
Application of Correlation Network Hub Prediction (CNHP). **(A)** Heatmap showing correlation between genes of T cell receptor signaling pathway upregulated in Chagas cardiomyopathy patients (CCC) and CD8+, CD4+, regulatory (Treg) and memory CD4+ (Tmem) T cells. **(C)** Heatmap showing correlation between genes of Natural-killer (NK) cell mediated cytotoxicity pathway upregulated in CCC and CD8+, CD4+ and mature NK cells. Immuno-Navigator was used to analysis the correlation of DEGs in heart tissue from CCC (GSE84796) and immune cells. Data are represented as correlation score (-log10 p value). In the color gradient bar, darker color indicates higher significant difference, while lighter color indicates lower significant difference. **(B, D)** represent the networks built between the pathways and cell types.

Given that the T-cell receptor signaling pathway appeared as the top hit of the overall DEG analysis in the heart (**Figure 3C**), we employed the Immuno-Navigator tool to predict what T-cell populations were associated with the upregulated genes. For this purpose, we examined the interaction between the genes of TCR signaling pathway and CD4+, CD8+, regulatory (Treg) and memory (Tmem) T cells. Interaction network hub prediction indicates that genes upregulated in TCR signaling pathway are mainly correlated with CD8+ T cells, followed by CD4+, regulatory (Treg) and memory (Tmem) T cells (**Figure 4A**). Network analysis showed greater number of interactions within the CD8 as compared to the CD4 compartment (**Figure 4B**; **Supplementary Figure 2**). We also performed interaction network between NK cell-mediated cytotoxicity pathway, which is upregulated in the heart of CCC (**Figure 3C**, third hit), with CD8+, CD4+ and mature NK cells, and observed that this pathway was mainly correlated with CD8+ T cells, followed by mature NK and CD4+ cells (**Figure 4C**). Although the inputted pathway was the NK cell-mediated cytotoxicity pathway, network analysis showed greater interaction with CD8+ cells even when compared with NK cells (**Figure 4D**; **Supplementary Figure 2**).

## 4 Discussion

Multi-omic studies have been conducted in distinct pathologies to elucidate immune and molecular mechanisms and to identify key drivers and signaling pathways underlying disease pathogenesis. In the current study, we used an integration of *in situ* transcriptomic data and circulating soluble mediator analysis from patients with CCC to obtain new insights into cardiomyopathy-driving mechanisms, and to ascertain the correspondence between the immune response observed in blood and tissue. We found a significant correspondence between circulating factors and their gene expression in the heart of CCC patients. In addition, we found similar signaling pathways mobilized in both compartments. We also observed that the main signaling pathway mobilized in the heart of CCC patients was the T-cell receptor signaling pathway and that the genes from this pathway were mainly correlated with CD8 cells, which displayed strong association with cytotoxic pathway genes. Collectively, these data provide novel information regarding immune molecules, their associated signaling pathways and potential targets associated with CCC pathology, while validating the blood as an important non-invasive source of information.

The association of increased inflammatory cytokines and chemokines with CCC has been shown by several authors (reviewed by Koh et al, 2023). Here, using a well-defined cohort of 84 patients, we confirmed this data and showed correlation of the inflammatory cytokines IL-6, IFN-gamma and TNF with cardiac dysfunction, as well as of IL-17 with better cardiac function. These correlations between cytokine plasma levels and cardiac function strengthens the association between systemic immune response and heart pathology. Anti-inflammatory cytokines such as IL-10 have been associated with the IND clinical form, while CCC display lower levels of this cytokine (reviewed in 11). It is hypothesized that the decrease in IL-10 expression may be related to the progression of the inflammation and, thus, disease evolution. However, longitudinal studies are needed to properly address this question.

Consistent with this phenotypic expression, Th17 and Th1/Th2 cells differentiation pathways were enriched in CCC heart tissue, similar to observed in previous study (19). Th17 cells, which can be induced by IL-1β and IL-6 (29), have been associated with a protective immune response in Chagas disease (30–33). This is likely due to the role of IL-17 in controlling parasite growth (34) and cardiac inflammation through the negative feedback of chemokines (30). Given the long-lasting nature of CCC, upregulation of Th17 differentiation pathway DEGs in CCC heart tissue may be a mechanism of modulating the inflammatory response to control fast tissue damage. Our study revealed an enrichment of the TNF signaling pathway in both the blood and heart tissue of CCC patients. Notably, we observed an elevated expression of TNFR1 in circulating T cells in prior studies (12, 35). In addition, the TNF/TNFR1 signaling cascade has previously been linked to the development of CD8-enriched myocarditis and the underlying pathophysiology during *T. cruzi* infection (36).

Previous studies have shown increased expression of CCL5, CXCL9, CXCL10, CCL17, CCL19 and their receptors in CCC myocardium (2, 37). Here we found increased levels of CCL5 and CXCL10 in the blood of Chagas patients, mirroring myocardium data. We also showed here that the chemokine signaling pathway is enriched in CCC tissue. Cunha-Neto et al. (2005) suggested a direct activity of these chemokines on cardiomyocytes, inducing the upregulation of genes involved in hypertrophy and heart failure (6), implicating chemokines in disease pathology, by a mechanism distinct and perhaps complementary to cell recruitment. Of note, the manuscript by Feng Li et al. showed that enrichment analysis revealed that DEGs in heart of patients with dilated cardiomyopathies were also involved in the immune-related pathological process (38).

A broad analysis of all DEG in the heart of CCC patients showed that TCR signaling pathway was upregulated. Canonical-correlation analysis indicated that these DEG generate an interconnected network and, our approach suggests that CCC patients exhibit an upregulation of genes involved in inflammatory immune response signaling pathways and an enrichment of biological processes that occur mainly in immune cells. In support of these observations, DEGs involved in TCR signaling pathway were correlated with CD8+ and CD4+ T cells, which showed an upregulation and interconnection of key genes in immunological pathways important to infection control and progression of CCC. Previous studies have demonstrated the predominance of CD8+ T cells in the myocardium of CCC, which are correlated to higher inflammation and fibrosis, as well as TNF expression (14, 38, 39). If, in one hand, CD8+ T cells are essential for the recognition of cells infected by *T. cruzi* and, consequently, for controlling the infection in the acute phase (40), on the other hand a massive cytotoxicity mediated by CD8+ T cells may contribute to heart tissue damage and progression of CCC (14, 41). Notably, molecules important for T-cell cytotoxicity, such as perforin, granzyme A and the Eomes transcription factor (42) are upregulated in the cardiac tissue and displays increased expression in circulating T cells of CCC patients (12, 35), corroborating previously suggested data of the critical role of these cells in the pathogenesis of CCC (12, 14, 38, 39). It is noteworthy to mention that the analysis of NK-derived cytotoxic genes displayed an association with CD8+ cells that was stronger than the one observed with NK cells themselves, implicating CD8 cells in disease pathology. Activated CD8+ cells were also observed in the peripheral blood of Chagas disease patients (12, 43–45).

Here, we observed an expressive correspondence of molecules analyzed in blood and heart, in a way that molecules that increased, decreased or did not change in one compartment, displayed the same behavior in another. This is even more striking when we consider the differences in the methods used for the analysis (soluble factors versus gene expression), and that the analyses were independently done in distinct patient groups.

The present study has some limitations. First, there is no transcriptome data from IND to compare with CCC. Although IND do not show clinical signs and symptoms of impairment of cardiac structure and function, the analysis of DEGs between distinct clinical forms of Chagas disease will provide relevant insight into the mechanisms that drive different clinical outcomes and disease progression. Second, transcriptome data considering patients with different degrees of cardiomyopathy is not available, and the data represents solely a spectrum of patients with severe disease, requiring heart transplantation. Finally, the correlation analysis of DEGs with different cells of immune system, performed in the Immuno-Navigator, is based on limited genetic data that may lead to biased results due to the plasticity of factors that predispose to different diseases. While these issues inspire future studies, this current study provides compelling evidence for further research about immune-related genes as modulators of cardiac damage and progression of Chagas heart disease.

**Figure 5:**
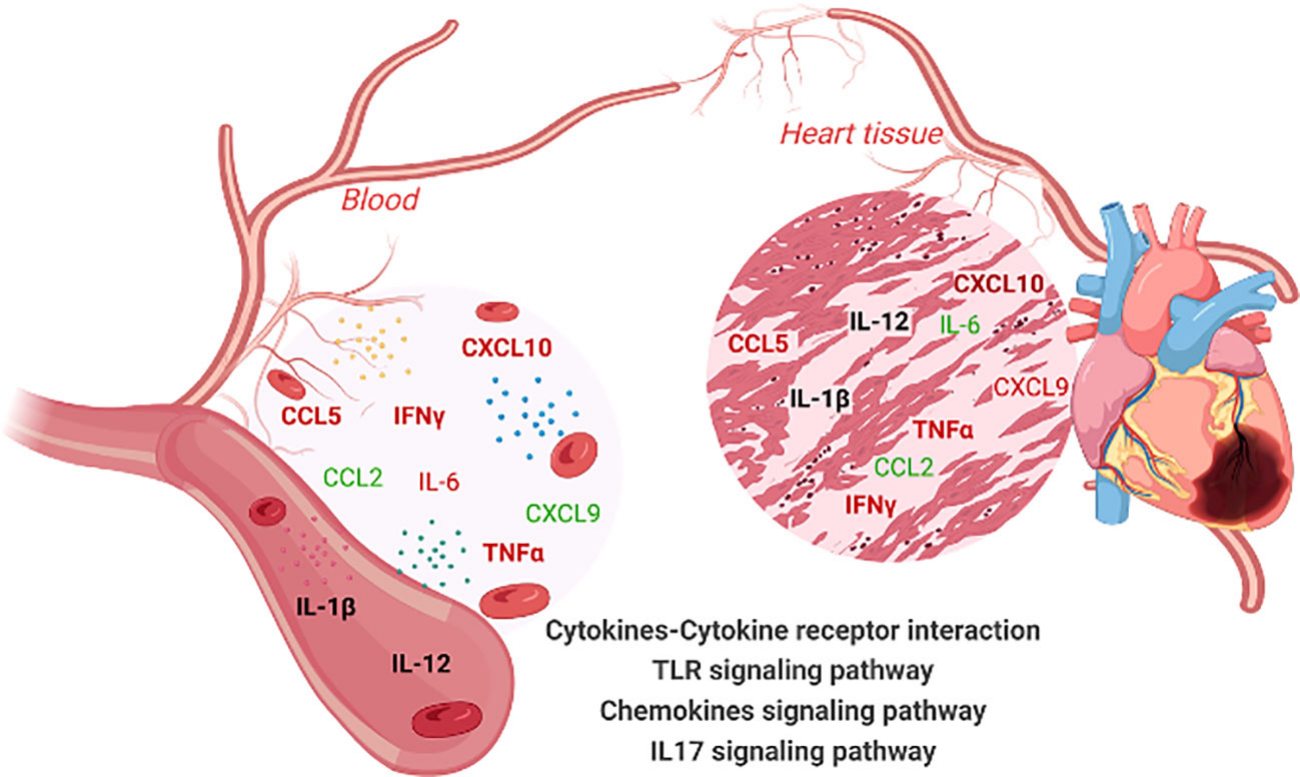
Overview of immune molecule interaction network in the blood and heart tissues of chronic Chagas disease (CCC) patients. This illustration summarizes the interaction network of immune molecules in the blood and heart tissue of patients with chronic Chagas disease (CCC). Elevated levels of IL-6, IFN-γ, TNF-α, CXCL10, CCL5 are observed in both the blood and heart tissue. Furthermore, the levels of IL-1β and IL12 remain unchanged in both compartments (blood and heart). The CCL2 chemokine is decreased in plasma and heart of CCC. Notably, an equivalence is observed between the levels of soluble factors in the plasma and their gene expression levels in the cardiac tissue of CCC patients. Importantly, these soluble mediators in the blood and their corresponding genes in the heart are implicated in the enrichment of the same signaling pathways: cytokine-cytokine receptor interaction, Toll-like receptor pathway, IL-17 signaling pathway and Chemokine signaling pathways. Molecules highlighted in bold indicate similar behavior in the blood and heart tissue. Color code: molecules highlighted in red are increased, in green are reduced and, in black, remained unchanged. Created with BioRender.com.

In summary, our systemic and integrative approach showed that DEG in cardiac tissue of CCC reflect the immunological profile observed at the systemic levels. The increased expression of circulating inflammatory cytokines and their genes in the plasma and heart tissue of CCC, as well as the association of circulating molecules with heart function, strengthens the association of inflammatory response with cardiac disease severity. This information is summarized in **Figure 5**. Importantly our study substantiates blood as a non-invasive source of information and suggests that the analysis of soluble mediators will support the identification and establishment of biomarkers of Chagas disease progression. This approach may also be extensive to other diseases where target organs are difficult to obtain.

## Data availability statement

The datasets presented in this study can be found in online repositories. The names of the repository/repositories and accession number(s) can be found in the article/**Supplementary Material**.

## Ethics statement

The studies involving humans were approved by Comitê de Ética da Universidade Federal de Minas Gerais - COEP. The studies were conducted in accordance with the local legislation and institutional requirements. The participants provided their written informed consent to participate in this study.

## Author contributions

TS-S: Conceptualization, Data curation, Formal Analysis, Investigation, Methodology, Visualization, Writing – original draft. EN: Data curation, Investigation, Writing – review & editing. MN: Investigation, Resources, Writing – review & editing. JG: Investigation, Resources, Writing – review & editing. KG: Investigation, Supervision, Writing – review & editing. WD: Conceptualization, Funding acquisition, Investigation, Methodology, Project administration, Resources, Supervision, Writing – original draft, Writing – review & editing. CK: Methodology, Writing – review & editing. ATC: Methodology, Writing – review & editing; SA: Investigation and Methodology.
