# Supplementary material for: Correlation of blood-based immune molecules with cardiac gene expression profiles reveals insights into Chagas cardiomyopathy pathogenesis
Source: Front Immunol. 2024 Feb 8;15:1338582. doi: 10.3389/fimmu.2024.1338582 (PMC10882095; doi:10.3389/fimmu.2024.1338582)
Supplement: Supplementary file 1 [file Presentation_1.pdf]

# *Supplementary Material*

# Correlation of Blood-Based Immune Molecules with Cardiac Gene Expression Profiles Reveals Insights into Chagas Cardiomyopathy Pathogenesis

*** Correspondence:**
Corresponding author: Walderez Ornelas Dutra
waldutra@gmail.com

**Supplementary Table 1**

| Donors | CTRL | | IND | | CCC | |
|---|---|---|---|---|---|---|
| **Sex** | F | 7 (58.3) | F | 10 (31.2) | F | 16 (40) |
| | M | 5 (41.6) | M | 22 (68.7) | M | 24 (60) |
| **Age (years)** | 31 ± 9 | | 46 ± 9.7 | | 51.4 ± 11.9 | |
| **Clinical Forms (n)** | 12 | | 32 | | 40 | |
| **LVEF** | >54 | | 65.6 ±4.3[a] | | 43.9 ±8.7[a] | |
| **LVDD** | <32 | | 47.8 ±4.9[a] | | 62.2 ± 6.1[a] | |

**Demographic and clinical data of the donors evaluated in the study.** Sex Variable were presented by n (%). Data are represented as means ± SD (Standard deviation). Same letters in different groups represent statically significant differences between them ($p<0.001$).

## Supplementary Table 2

| Molecules | Blood | | Heart | |
|---|---|---|---|---|
| | CTRL | CCC | CTRL | CCC |
| NGF | 27.8 ± 11.8 | 28.9±22.2 | 0.65±0.8 | -0.11±0.2** |
| CCL27 | 95.3±55 | 21.5±6.2* | 0.03±0.8 | 0.26±0.5** |
| CCL11 | 508.5±36.3 | 935.8±621.5* | -0.2±2.8 | 0.34±0.9 |
| **FGF** | **23±7.7** | **25.7±7.8** | **0.2±0.7** | **-0.18±0.9** |
| G-CSF | 95.9±71.6 | 316.2±54.5* | NA | NA |
| GM-CSF | 27.2±14.7 | 27.5±20* | NA | NA |
| CXCL1 | 55.6±15.4 | 66.6±22.7 | 1.2±1.9 | -0.49±1.3** |
| HGF | 97.8±49.6 | 188.4±107.4* | 0.11±1.46 | -0.13±0.7 |
| **IFNα** | **22.4±10.3** | **25.3±16.5** | **-0.13±0.5** | **-0.46±0.7** |
| IFNγ | 41.38±14.5 | 58.53±26.5 | -0.65±0.07 | 1.33±0.28** |
| **IL1α** | **23±6.2** | **25.1±8.9** | **0.04±0.3** | **0.26±0.9** |
| IL1β | 33.1±14.8 | 44.3±22.8 | 1.2±0.5 | 0.06±0.01** |
| IL1RA | 100.5±214 | 245.6±367.1* | 0.08±0.9 | -0.03±0.6** |
| IL2 | 23.6±13.7 | 19.8±14.8 | -0.42±0.3 | 0.39±0.5** |
| IL2RA | 204.9±127.8 | 402.4±221.6 | -0.78±0.9 | 0.39±1.3** |
| **IL3** | **27.3±14.2** | **27.4±15.8** | **0.24±0.7** | **0.34±0.5** |
| **IL4** | **39.7±11.6** | **55.1±21.8*** | **-0.70±0.3** | **0.52±0.7**** |
| **IL5** | **29.1±17.1** | **29.8±24.1** | **0.05±0.6** | **0.41±0.9** |
| IL6 | 30.7±19.2 | 32.7±30.1 | 1.5±0.03 | -0.32±0.001** |
| IL7 | 60.8±65.5 | 74±109.7 | -1.47±0.9 | 1.17±0.8** |
| IL8 | 37.9±21.4 | 159.8±21.7* | 1.22±2.2 | 0.28±1.06 |
| IL9 | 150.7±44.9 | 164±5.7* | 0.13±0.3 | 0.38±0.7 |
| **IL10** | **37.4±29.9** | **32.5±31.1** | **-0.15±0.03** | **0.06±0.02** |
| **IL12p70** | **40.6±41.7** | **38.8±45.5** | **-0.26±0.02** | **-0.003±0.002** |
| **IL12p40** | **20.3±11.4** | **19.2±12** | **-0.26±0.3** | **0.15±0.5** |
| **IL13** | **25.7±23** | **17±10.5** | **-0.07±0.7** | **0.13±0.4** |
| IL15 | 27.3±12.7 | 28±23.4 | -0.32±0.5 | 0.22±0.4** |
| IL16 | 129.6±105.5 | 262.153.2 | -1.57±0.4 | 0.94±0.6** |
| **IL17A** | **41.7±42.3** | **47.6±41.2** | **0.5±0.02** | **0.03±0.003** |

| | | | | |
|---|---|---|---|---|
| IL18 | 46.5±21.2 | 59.4±40.5 | -1.25±0.5 | 1.02±1.1** |
| **CXCL10** | **382.7±246.1** | **1407±537*** | **-3.23±1.2** | **0.88±0.27**** |
| **LIF** | **21.3±11.4** | **25.1±14.2** | **0.21±1** | **-0.37±1.3** |
| M-CSF | 59.6±28.5 | 124.7±54.8* | NA | NA |
| MCP1 | 71.6±43.9 | 94.9±59.2 | 1.8±0.7 | -0.29±0.73** |
| **MCP3** | **20.2±12.7** | **21.8±13.5** | **0.63±1.5** | **-0.21±0.5** |
| **MIF** | **220.7±123.8** | **379.6±142.9*** | **-0.08±0.3** | **-0.001±0.2** |
| **CXCL9** | **267.84±146.3** | **3033±636*** | **-4.5±0.93** | **1.49±0.37**** |
| **CCL3** | **42.3±24.3** | **144.8±110*** | **-2.12±1.7** | **0.47±0.6**** |
| **CCL4** | **480.2±172.4** | **606±204*** | **-3.87±1.3** | **0.99±0.7**** |
| **PDGF-bb** | **92±52.2** | **97.1±80.9** | **0.08±0.8** | **-0.19±0.9** |
| CCL5 | 2038.2±657.1 | 2016±988.8 | -4±2.3 | 1.11±0.29** |
| SCF | 72.8±25.7 | 98.9±41.7* | NA | NA |
| SCGF-b | 960.4±282.4 | 955.3±476.1 | NA | NA |
| **CXCL12** | **281.7±76.5** | **288.3±70.7** | **0.04±0.2** | **-0.27±0.5** |
| TNFα | 23.4±13.6 | 18.7±16.1* | -0.79±0.17 | 0.89±0.17** |
| **TNFβ** | **382.2±76.3** | **416.7±99.5** | **0.05±0.4** | **-0.63±0.7** |
| **TRAIL** | **60.5±35** | **162.9±407.9** | **0.63±0.1** | **-0.21±0.1** |
| **VEGF** | **27.3±13.4** | **48.6±92.8** | **-0.08±0.3** | **-0.001±0.003** |

**Plasma and heart tissue levels of immune molecules in controls (CTRL) and cardiac (CCC) Chagas patients.** Soluble mediators were measured using the Bio-Plex ProTM Human Cytokine Standard kit and results were expressed in MFI. Differentially expressed genes of human left ventricular free wall heart tissue from CCC and CTRL (GSE84796) were analyzed as described in material and methods. NA: Not available. Molecules with similar behavior between blood and heart tissue (either increased, decreased or did not change in both compartments) were highlighted in bold. Data is presented as mean ± SD. *Indicates a statistically significant difference ($p< 0.05$) between the CTRL and CCC groups in blood. ** Indicates a statistically significant difference ($p< 0.05$) between the CTRL and CCC groups in heart tissue.

## Supplementary Figure 1

A

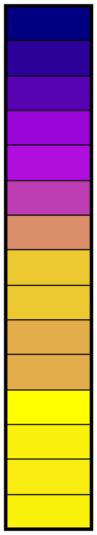

| GO: Biologic processes | Total | Expected | Hits | P. Value | FDR |
|---|---|---|---|---|---|
| **Defense response** | 1510 | 23.1 | 100 | 9.93e-41 | 8.15e-38 |
| **Immune response** | 1430 | 21.9 | 95 | 1.78e-38 | 7.29e-36 |
| **Cytokine-mediated signaling pathway** | 374 | 5.73 | 53 | 2.08e-36 | 5.7e-34 |
| **Inflammatory response** | 569 | 8.72 | 60 | 6.35e-34 | 1.3e-31 |
| Regulation of defense response | 519 | 7.95 | 56 | 4.29e-32 | 7.04e-30 |
| Immune system process | 2720 | 41.6 | 119 | 7.07e-32 | 9.66e-30 |
| **Multi organism process** | 1710 | 26.2 | 90 | 2.93e-28 | 2.67e-26 |
| **Regulation of I-κB kinase/NF-κB cascade** | 210 | 3.22 | 36 | 1.16e-27 | 9.32e-26 |
| Positive regulation of immune system process | 739 | 11.3 | 60 | 1.25e-27 | 9.32e-26 |
| **I-κB kinase/NF-κB cascade** | 246 | 3.77 | 38 | 1.92e-27 | 1.27e-25 |
| **Innate immune response** | 638 | 9.78 | 56 | 2.02e-27 | 1.27e-25 |
| **Intracellular protein kinase cascade** | 1140 | 17.4 | 72 | 9.67e-27 | 5.66e-25 |
| **Transcription from RNA pol II promoter** | 1930 | 29.6 | 92 | 1.06e-25 | 5.43e-24 |
| Response to wounding | 1310 | 20.1 | 75 | 4.27e-25 | 2.06e-23 |
| Regulation of transcription from RNA pol II promoter | 1610 | 24.6 | 82 | 2.17e-24 | 9.37e-23 |

B

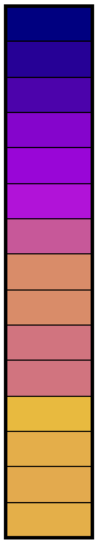

| GO: Biologic processes | Total | Expected | Hits | P. Value | FDR |
|---|---|---|---|---|---|
| **Immune response** | 1430 | 16.6 | 77 | 7.94e-34 | 6.51e-31 |
| **Defense response** | 1510 | 17.5 | 78 | 4.81e-33 | 1.97e-30 |
| **Inflammatory response** | 569 | 6.61 | 50 | 1.52e-30 | 2.5e-28 |
| Regulation of immune system process | 1190 | 13.9 | 67 | 8.32e-30 | 9.75e-28 |
| **Cytokine-mediated signaling pathway** | 374 | 4.35 | 41 | 1.17e-28 | 1.2e-26 |
| **Regulation of I-kappaB kinase/NF-kappaB cascade** | 210 | 2.44 | 30 | 2.11e-24 | 1.57e-22 |
| **Intracellular protein kinase cascade** | 1140 | 13.2 | 59 | 4.28e-24 | 2.7e-22 |
| **I-κB kinase/NF-κB cascade** | 246 | 2.86 | 31 | 1.64e-23 | 9.62e-22 |
| Innate immune response | 638 | 7.41 | 45 | 2.29e-23 | 1.25e-21 |
| **Response to wounding** | 1310 | 15.3 | 61 | 1.9e-22 | 9.71e-21 |
| **Multi-organism process** | 1710 | 19.8 | 69 | 2.8e-22 | 1.35e-20 |
| Response to other organism | 716 | 8.32 | 45 | 2.49e-21 | 1.08e-19 |
| Response to biotic stimulus | 749 | 8.7 | 45 | 1.51e-20 | 6.2e-19 |
| Regulation of cytokine production | 513 | 5.96 | 38 | 2.07e-20 | 8.08e-19 |
| **Transcription from RNA pol II promoter** | 1930 | 22.4 | 69 | 2.78e-19 | 1.04e-17 |

-log10 (adjusted p-value)

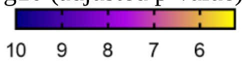

**Gene ontology of biologic processes enriched in (A)** peripheral blood and **(B)** heart tissue from CCC. Soluble mediators differentially modulated in the blood and DEGs of human left ventricular free wall heart tissue from CCC (GSE84796) were used to perform biologic processes (top 15) enriched by NetworkAnalystic.ca. in the color gradient bar, dark blue indicates higher significant difference, while yellow denote lower difference DEGS: Differentially expressed genes.

## Supplementary Figure 2

Figure 4 B

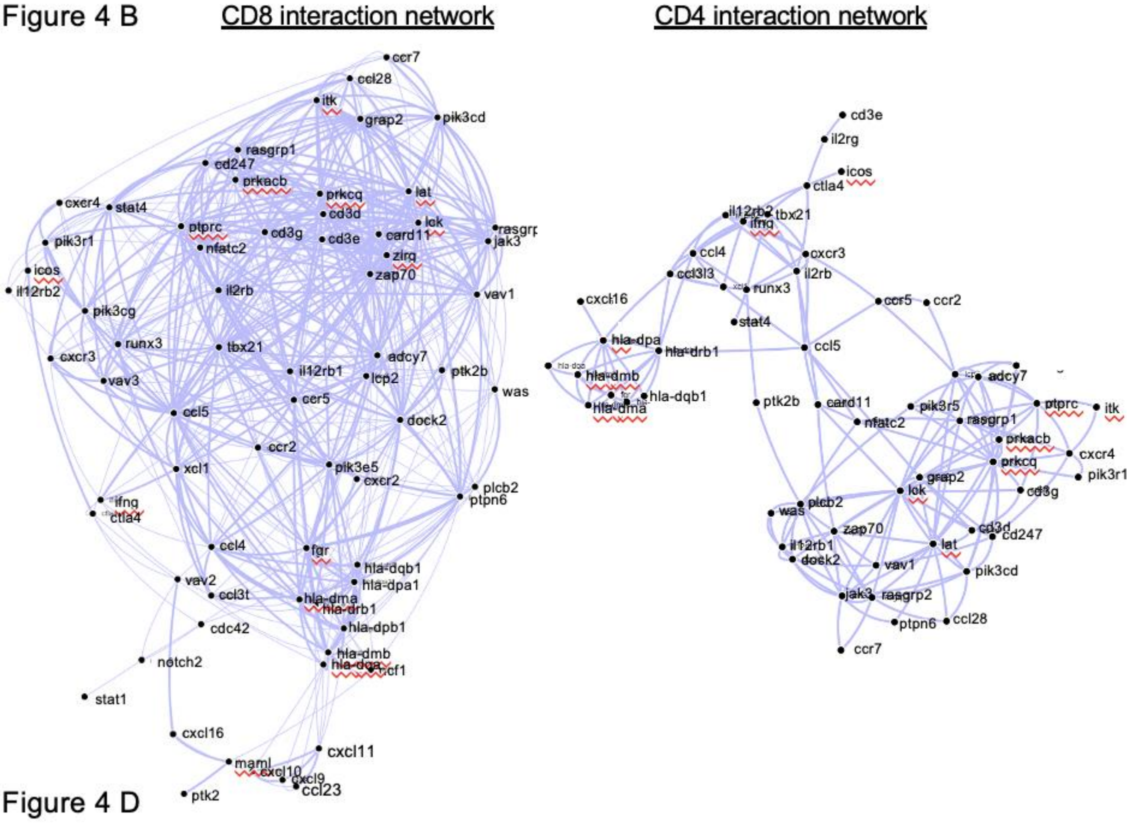


Figure 4 D

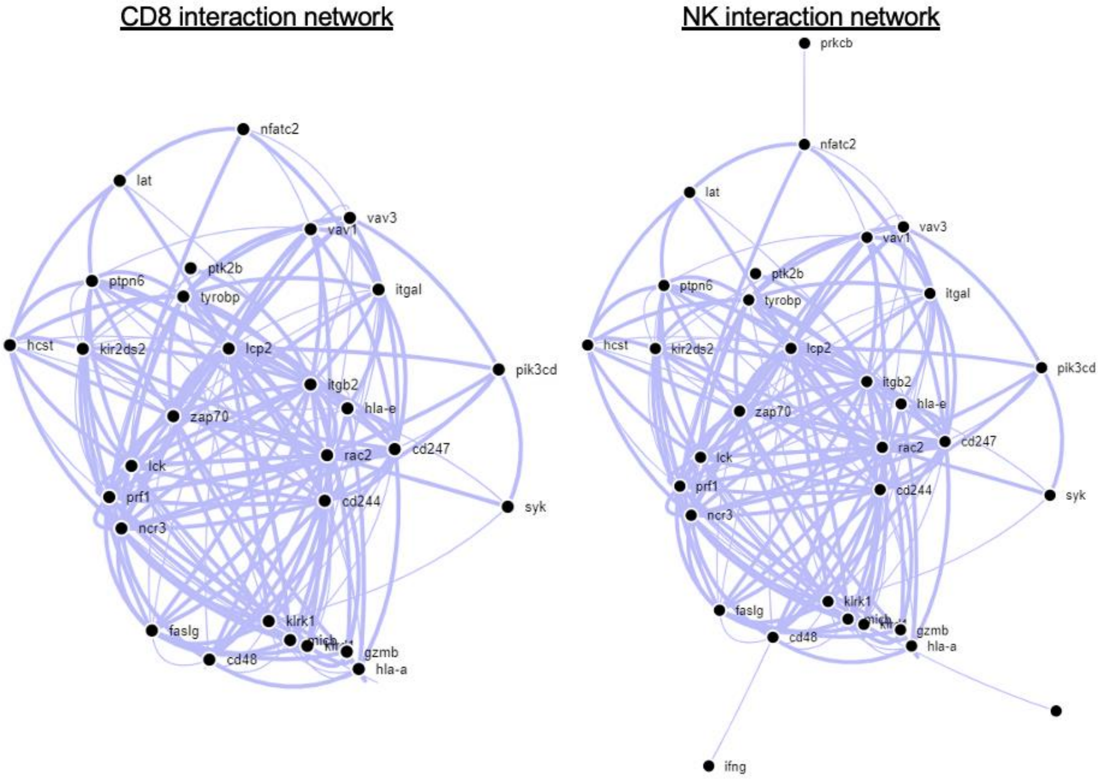

**Details of panels (B)** and **(D)** from Figure 4 of the manuscript, identifying each node present in the figures. The decription is the same as in the original legend, only here it is possible to verify the molecules.
